# Supplementary material for: Roles of adipose-derived stem cells and derived exosomes in therapeutic applications to testicular injury caused by cisplatin
Source: PLoS One. 2024 Feb 5;19(2):e0297076. doi: 10.1371/journal.pone.0297076 (PMC10843130; doi:10.1371/journal.pone.0297076)
Supplement: Attachment — Submitted filename: PONE-D-23-25567_reviewer.pdf [file pone.0297076.s001.pdf]

PLOS ONE

Adipose-derived stem cells and derived exosomes may alleviate the testicular injury caused by cisplatin.

--Manuscript Draft--

**Manuscript Number:**	PONE-D-23-25567
**Article Type:**	Research Article
**Full Title:**	Adipose-derived stem cells and derived exosomes may alleviate the testicular injury caused by cisplatin.
**Short Title:**	Adipose-derived stem cells and derived exosomes may alleviate the testicular injury caused by cisplatin.
**Corresponding Author:**	Shixuan wu<br>The First Affiliated Hospital of Zhengzhou University<br>zhengzhou, CHINA
**Keywords:**	Keywords：Adipose-derived stem cells，derived exosomes，cisplatin，testicular injury.
**Abstract:**	We aimed to explore the potential treatment of adipose-derived stem cells and derived exosomes for testicular injury caused by cisplatin.Adipose-derived stem cells and derived exosomes were identified and extracted to treat the rat model with adipose-derived stem cells and derived exosomes. Then the immunohistochemistry and E[illegible]A assay were used to detect the potential treatment of adipose-derived stem cells and derived exosomes.We found that adipose-derived stem cells and derived exosomes significantly improved the testicular tissue damage, increased the number of germ cells, and improved the arrangement of the seminiferous tubules. The levels of malondialdehyde and testosterone were also improved. We speculated that adipose-derived stem cells and derived exosomes may alleviate the testicular injury caused by cisplatin.
**Order of Authors:**	Shixuan wu
**Additional Information:**	
**Question**	**Response**
**Financial Disclosure**<br><br>Enter a financial disclosure statement that describes the sources of funding for the work included in this submission. Review the submission guidelines for detailed requirements. View published research articles from *PLOS ONE* for specific examples.<br><br>This statement is required for submission and **will appear in the published article** if the submission is accepted. Please make sure it is accurate.	Rui Wang was supported by the Henan Provincial Health Care Commission Provincial Ministry Project Fund (LHGJ20190276).

Powered by Editorial Manager® and ProduXion Manager® from Aries Systems Corporation

**Unfunded studies**<br>Enter: *The author(s) received no specific funding for this work.*<br><br>**Funded studies**<br>Enter a statement with the following details:<br>• Initials of the authors who received each award<br>• Grant numbers awarded to each author<br>• The full name of each funder<br>• URL of each funder website<br>• Did the sponsors or funders play any role in the study design, data collection and analysis, decision to publish, or preparation of the manuscript?<br>• **NO** - Include this sentence at the end of your statement: *The funders had no role in study design, data collection and analysis, decision to publish, or preparation of the manuscript.*<br>• **YES** - Specify the role(s) played.<br><br>* typeset	
**Competing Interests**<br><br>Use the instructions below to enter a competing interest statement for this submission. On behalf of all authors, disclose any competing interests that could be perceived to bias this work—acknowledging all financial support and any other relevant financial or non-financial competing interests.<br><br>This statement is required for submission and **will appear in the published article** if the submission is accepted. Please make sure it is accurate and that any funding sources listed in your Funding Information later in the submission form are also declared in your Financial Disclosure statement.<br><br>View published research articles from *PLOS ONE* for specific examples.	The authors have declared that no competing interests exist.

Powered by Editorial Manager® and ProduXion Manager® from Aries Systems Corporation

**NO authors have competing interests**<br><br>Enter: *The authors have declared that no competing interests exist.*<br><br>**Authors with competing interests**<br><br>Enter competing interest details beginning with this statement:<br><br>*I have read the journal's policy and the authors of this manuscript have the following competing interests: [insert competing interests here]*<br><br>* typeset	
**Ethics Statement**<br><br>Enter an ethics statement for this submission. This statement is required if the study involved:<br><br>• Human participants<br>• Human specimens or tissue<br>• Vertebrate animals or cephalopods<br>• Vertebrate embryos or tissues<br>• Field research<br><br>Write "N/A" if the submission does not require an ethics statement.<br><br>General guidance is provided below. Consult the submission guidelines for detailed instructions. **Make sure that all information entered here is included in the Methods section of the manuscript.**	The authors are accountable for all aspects of the work in ensuring that questions related to the accuracy or integrity of any part of the work are appropriately investigated and resolved. The study was conducted in accordance with the Declaration of Helsinki (as revised in 2013). Experiments were performed under a project license(No.ZZU-LAC20190125) granted by the Animal Ethical and Welfare Committee, in compliance with the ethics committee of the First Affiliated Hospital of Zhengzhou University.

Powered by Editorial Manager® and ProduXion Manager® from Aries Systems Corporation

**Format for specific study types**<br><br>**Human Subject Research (involving human participants and/or tissue)**<br>• Give the name of the institutional review board or ethics committee that approved the study<br>• Include the approval number and/or a statement indicating approval of this research<br>• Indicate the form of consent obtained (written/oral) or the reason that consent was not obtained (e.g. the data were analyzed anonymously)<br><br>**Animal Research (involving vertebrate animals, embryos or tissues)**<br>• Provide the name of the Institutional Animal Care and Use Committee (IACUC) or other relevant ethics board that reviewed the study protocol, and indicate whether they approved this research or granted a formal waiver of ethical approval<br>• Include an approval number if one was obtained<br>• If the study involved *non-human primates*, add *additional details* about animal welfare and steps taken to ameliorate suffering<br>• If anesthesia, euthanasia, or any kind of animal sacrifice is part of the study, include briefly which substances and/or methods were applied<br><br>**Field Research**<br><br>Include the following details if this study involves the collection of plant, animal, or other materials from a natural setting:<br>• Field permit number<br>• Name of the institution or relevant body that granted permission	
**Data Availability**<br><br>Authors are required to make all data underlying the findings described fully available, without restriction, and from the time of publication. PLOS allows rare exceptions to address legal and ethical concerns. See the PLOS Data Policy and FAQ for detailed information.	Yes - all data are fully available without restriction

A Data Availability Statement describing where the data can be found is required at submission. Your answers to this question constitute the Data Availability Statement and **will be published in the article**, if accepted.<br><br>**Important:** Stating 'data available on request from the author' is not sufficient. If your data are only available upon request, select 'No' for the first question and explain your exceptional situation in the text box.<br><br>Do the authors confirm that all data underlying the findings described in their manuscript are fully available without restriction?	
**Describe where the data may be found in full sentences. If you are copying our sample text, replace any instances of XXX with the appropriate details.**<br><br>• If the data are **held or will be held in a public repository**, include URLs, accession numbers or DOIs. If this information will only be available after acceptance, indicate this by ticking the box below. For example: *All XXX files are available from the XXX database (accession number(s) XXX, XXX.)*.<br>• If the data are all contained **within the manuscript and/or Supporting Information files**, enter the following: *All relevant data are within the manuscript and its Supporting Information files.*<br>• If neither of these applies but you are able to provide **details of access elsewhere**, with or without limitations, please do so. For example:<br><br>*Data cannot be shared publicly because of [XXX]. Data are available from the XXX Institutional Data Access / Ethics Committee (contact via XXX) for researchers who meet the criteria for access to confidential data.*<br><br>*The data underlying the results presented in the study are available from (include the name of the third party*	All relevant data are within the manuscript and its Supporting Information files.

and contact information or URL).<br>• This text is appropriate if the data are owned by a third party and authors do not have permission to share the data.<br><br>* typeset	
Additional data availability information:	

Click here to access/download;Manuscript;manuscript.docx

1. # Adipose-derived stem cells and derived exosomes may alleviate the testicular injury caused by cisplatin.

2. **Shixuan Wu[1#] Kunlong Lv[1] Tao Zheng[1] Tianbiao Zhang[1] Yonghao Nan[1] Rui Wang[1*]**

3. **institutional affiliations:**[1]Department of Andrology, The First Affiliated Hospital of Zhengzhou University, Zhengzhou, China.

4. **Correspondence: *Rui Wang**

5. **Address:** Department of Andrology, The First Affiliated Hospital of Zhengzhou University, Zhengzhou, China.

6. **E-mail:** wang998rui@21cn.com

7. **Telephone numbers:**13733872598

8. **Running Title:** Effect of ADSC-Ex on testicular injury

9. **Word Count：**4533

10. **Number of Figures and Tables:**6

11.

Contributions: (I) Conception and design:Shixuan Wu; (II) Administrative support: Rui Wang; (III) Provision of study materials or patients:Shixuan Wu,Tao Zheng ; (IV) Collection and assembly of data: Tao Zheng,Tianbiao Zhang; (V) Data analysis and interpretation: Shixuan Wu,Yonghao Nan,Kunlong Lv; (VI) Manuscript writing: All authors; (VII) Final approval of manuscript: All authors.

1.Introduction:

Cisplatin is a widely used chemotherapy drug that can cause testicular damage, including germ cell loss, Leydig cell dysfunction, and damage to the blood-testis barrier (Abdel-Latif et al., 2022). This can lead to infertility and hormonal imbalances in male cancer survivors (Kohsaka et al., 2020).

Adipose-derived stem cells (ADSCs) are a type of mesenchymal stem cell that can be isolated from adipose tissue .(Qin et al., 2023) ADSCs have the ability to differentiate into multiple cell types, including adipocytes, osteoblasts, chondrocytes, and myocytes (Elsharkawi et al., 2023). In addition, ADSCs secrete various growth factors and cytokines that promote tissue repair and regeneration (Gecys et al., 2023). Exosomes are small membrane-bound vesicles that can transport proteins, nucleic acids, and other molecules between cells (Phelps et al., 2022). Exosomes derived from ADSCs, known as ADSC-Ex, have been shown to have similar therapeutic effects as ADSCs themselves (Wang et al., 2022). ADSC-Ex can promote tissue repair and regeneration, reduce inflammation, and modulate immune responses (Xia et al., 2022).

In recent years, ADSCs and ADSC-Ex have been investigated for their therapeutic potential in various diseases and conditions, including wound healing, tissue regeneration, autoimmune diseases, and neurological disorders (Gecys et al., 2023; Li et al., 2023). In preclinical studies, ADSCs and ADSC-[illegible]s have shown promising results in improving tissue function and reducing

inflammation in animal models of these diseases (Simao et al., 2023).

However, few studies reported the effect of ADSC-Ex treatment on testicular injury. In this study, we aimed to extract the ADSC-Ex and explore the impact of ADSC-Ex on rat models with testicular injury.

2.Methods:

2.1. Model establishment

The animal model of testicular cell damage was established by intraperitoneal injection of a 10mg/kg cisplatin solution. Three days after modeling, the testicular cell damage model + cell therapy group received a tail vein injection of 3*10^6 CM-DiI-labeled ADSCs cells, while the testicular cell damage model + Ex group received a tail vein injection of 100μg of ADSC-Ex The normal control group and the testicular cell damage model group received an equal volume of saline via tail vein injection. Serum and bilateral testes were collected for pathological examination 24 hours after extracellular vesicle injection.

2.2. Cell identification

Six-week-old SD rats were disinfected with 75% alcohol for 3 minutes. The rats were then placed on an ice plate in a laminar flow hood, and subcutaneous white adipose tissue was quickly removed from the inguinal area of the rats. Visible blood vessels and fibrous parts were removed from the adipose tissue, which was then washed three times with PBS, cut into small pieces, and digested with 0.2% collagenase I at 4°C overnight. The next day, the tissue was further digested at 37°C for 20 minutes with shaking every 5 minutes. The

digestion was stopped by adding a complete culture medium containing 10% FBS. The tissue digestion mixture was filtered through a 70-μm cell strainer, and the filtrate was collected and centrifuged at 1000 rpm for 5 minutes. The supernatant was discarded, and the cell pellet was resuspended in a fresh culture medium and cultured in a culture flask. The third passage of cells was collected by removing the culture medium and washing the cells twice with 1×PBS. The cells were then digested with 0.25% trypsin (containing 0.02% EDTA) until they became round. The digestion was stopped by adding a culture medium, and the cell suspension was collected and centrifuged at 1000 rpm for 3 minutes. The supernatant was discarded, and the cell pellet was resuspended in PBS and centrifuged again before being analyzed by flow cytometry.

2.3.Exosome extraction:

Thaw the sample at 37°C. Transfer the sample to a new centrifuge tube and centrifuge at 2000 × g, 4°C for 30 minutes. Carefully transfer the supernatant to a new centrifuge tube and centrifuge again at 10,000 × g, 4°C for 45 minutes to remove larger vesicles. Collect the supernatant, filter it through a 0.45 μm membrane, and collect the filtrate. Transfer the filtrate to a new centrifuge tube and centrifuge at 100,000 × g, 4°C for 70 minutes using an ultracentrifuge rotor. Discard the supernatant, resuspend the pellet in 10 mL of pre-cooled 1×PBS, and centrifuge again at 100,000 × g, 4°C for 70 minutes using an ultracentrifuge rotor. Discard the supernatant, resuspend the pellet in 100 μL of pre-cooled 1×PBS, and take 20 μL for electron microscopy, 10 μL for particle size analysis,

and 20 μL for fluorescence. The remaining extracellular vesicles are stored at -80°C.

2.4. Flow Cytometry:

Cells were collected, centrifuged to remove the supernatant, and washed once with 1 mL of PBS at 1500 rpm for 5 min. The cells were divided into six tubes, one tube being the negative control, and the other five tubes were added with 5 μL of CD29 PE, CD45 PERCP/CY5.5, CD90 FITC, CD34 PE, and CD11b/c FITC antibodies, respectively. After gentle mixing, the tubes were incubated at room temperature in the dark for 20 min. Then, 1 mL of PBS was added, the tubes were centrifuged at 1500 rpm for 5 min, and the supernatant was discarded. This step was repeated once. The cells were resuspended in 500 μL of PBS and analyzed by flow cytometry.

2.5. Evaluation of ADCS-Ex

Transmission electron microscopy observation of extracellular vesicle samples: 10 μL of extracellular vesicles were taken and dropped onto a copper grid for 1 min. The excess liquid was removed with filter paper. 10 μL of uranyl acetate was added to the copper grid for 1 min, and the excess liquid was removed with filter paper. The copper grid was air-dried for several minutes and observed by transmission electron microscopy to obtain the imaging results. Fluorescent labeling and nano flow cytometry detection of extracellular vesicle samples: 20 μL of extracellular vesicles were diluted to 60 μL, and 30 μL of the diluted extracellular vesicles were added to 20 μL of fluorescently labeled antibodies

(CD9, CD63), mixed, and incubated at 37°C in the dark for 30 min. 1 mL of pre-cooled PBS was added, and the tubes were centrifuged at 110,000 × g at 4°C for 70 min. The supernatant was carefully removed, and 1 mL of pre-cooled PBS was added. The tubes were centrifuged again at 110,000 × g at 4°C for 70 min. The supernatant was carefully removed, and the pellets were resuspended in 50 μL of pre-cooled 1×PBS. The instrument performance was tested with standard samples before extracellular vesicle samples were analyzed. Gradient dilution was performed to avoid sample blockage in the sample needle. The protein index results were obtained by detecting the samples using the NanoFCM instrument.

2.6. Immunohistochemistry:

Tissue sections were baked in a 65°C oven for 2 hours. The sections were deparaffinized with xylene for 10 minutes, followed by another 10 minutes in fresh xylene. The sections were then sequentially placed in 100% ethanol, 100% ethanol, 95% ethanol, 80% ethanol, and purified water for 5 minutes each. The sections were placed in a repair box and antigen retrieval was performed by adding antigen retrieval solution (citrate buffer) and heating in a pressure cooker until automatic release of pressure. After 2 minutes, the box was removed from the heat source and allowed to cool naturally. The antigen retrieval solution was discarded, and the sections were washed with PBS. The sections were then treated with 0.5% Triton X-100 (PBS solution) at room temperature for 20 minutes. The sections were washed with PBS for 3 times,

each for 5 minutes, and the surrounding PBS was removed by blotting with absorbent paper. 5% BSA was added to the sections and incubated at 37°C for 30 minutes to block non-specific binding. The excess blocking solution around the tissue was removed by blotting with absorbent paper, and the sections were not washed. A diluted primary antibody (c-Kit, 1:200) was added to each slide, and the slides were placed in a humid box and incubated at 4°C. The wet box incubated at 4°C overnight was taken out and left at room temperature for 45 minutes. The slides were washed with PBS for 3 times, each for 5 minutes, and then incubated with horseradish peroxidase-labeled goat anti-rabbit IgG (H+L) (1:100) at 37°C for 30 minutes. The slides were washed with PBS thoroughly. DAB was used for color development for 5-10 minutes, and the staining intensity was observed under a microscope. The slides were washed with PBS or tap water for 1 minute, followed by counterstaining with hematoxylin for 3 minutes and differentiation with hydrochloric acid alcohol until blue. The slides were washed with tap water for 1 minute, dehydrated, mounted, and examined under a microscope.

2.7. Histopathological evaluation

Testicular tissues were obtained and subjected to dehydration with 70%, 80%, and 90% ethanol solutions, followed by immersion in a mixture of equal volume of pure alcohol and xylene for 15 minutes, xylene I for 15 minutes, xylene II until it is transparent. The tissues were then placed in a mixture of half xylene and half paraffin wax for 15 minutes, followed by immersion in paraffin I and paraffin

II until transparent for 50-60 minutes each. The tissues were embedded in paraffin and sectioned. The paraffin sections were subjected to baking, deparaffinization, and hydration. The slices that have been distilled in distilled water were then stained with hematoxylin in a solution of distilled water for 3 minutes, differentiated in hydrochloric acid ethanol differentiation solution for 15 seconds, rinsed with tap water, put in a blueing solution for 15 seconds, rinsed with tap water, stained with eosin for 3 minutes, rinsed with water, dehydrated, cleared, sealed, and observed under a microscope. ImageJ software was used to measure the thickness of the epithelium of the seminiferous tubules and the diameter of the seminiferous tubules. To evaluate the degree of testicular damage and spermatogenic ability, the Mean Testicular Biopsy Score (MTBS) (Johnsen, 1970) was used. The score for each small tube was 1-10, based on the germ cell capacity of different tissues (table 1). A microscope magnified to 400 times was used to evaluate 20 seminiferous tubules in each group to assess the thickness of the epithelium of the seminiferous tubules and the diameter of the seminiferous tubules.

2.8. ELISA detection:

Standard wells, blank wells, and sample wells were set up. The standard wells were added with 100μL of standard product diluted by a certain ratio, the blank wells were added with 100μL of a standard product and sample dilution, and the remaining wells were added with 100μL of the test sample. The enzyme-labeled plate was covered and incubated at 37°C for 90 minutes. The liquid in

the wells was discarded without washing. Each well was added with 100μL of biotinylated antibody working solution, and the enzyme-labeled plate was covered and incubated at 37°C for 1 hour. The liquid in the wells was discarded, and the plate was blotted dry on clean absorbent paper. Each well was added with 350μL of washing solution, soaked for 1 minute, and the liquid in the enzyme-labeled plate was sucked or shaken off and blotted dry. This washing step was repeated 3 times. After washing, the plate should be used immediately for the next step and should not be allowed to dry. Each well was added with 100μL of enzyme conjugate working solution, and the enzyme-labeled plate was covered and incubated at 37°C for 30 minutes. The liquid in the wells was discarded, and the plate was washed 5 times, following the same method as in step 3. Each well was added with 90 μL of substrate solution (TMB), and the enzyme-labeled plate was covered and incubated at 37°C for about 15 minutes in the dark. The enzyme-labeled instrument was preheated 15 minutes in advance. Each well was added with 50 μL of stop solution to terminate the reaction. Note: The order of adding the stop solution should be as consistent as possible with that of the substrate solution. The optical density (OD value) of each well was immediately measured at 450 nm wavelength using an enzyme-labeled instrument.

2.9. Statistical analysis:

All data were analyzed using SPSS 22.0 and presented as mean ± SD. One-way ANOVA was used to analyze the significant differences between groups,

with P<0.05 as the criterion for statistical significance.

3.Results:

3.1. Models Validation:

The results, as shown in Figure 1, revealed that the pathological results (Figure 1A) showed a significant decrease in the layer of germ cells and a sparse arrangement of the seminiferous tubules in the testicular tissue of the model group compared to the control group. The immunohistochemical results (Figure 1B) showed a significant decrease in the expression level of c-Kit in the model group compared to the control group. The ELISA results (Figure 1C) showed a significant increase in the levels of follicle-stimulating hormone (FSH) and luteinizing hormone (LH), a significant decrease in the level of testosterone (T), and the differences were statistically significant (P<0.05).

3.2. Identification of ADSCs cells:

The results, as shown in Figure 2, revealed that the flow cytometry analysis showed positive expression of CD29 and CD90 and negative expression of CD11b, CD34, and CD45 in the cells (Figure 2A). The Alizarin Red staining indicated that the cells had the ability of osteogenic differentiation, suggesting the successful isolation of adipose-derived stem cells (Figure 2B).

3.3. Evaluation of ADSC-Ex:

Identification of ADSC-Ex through TEM, NTA and nanoflow cytometry analysis is shown in Figure 3. The morphology and size of ADSC-Ex were evaluated using TEM (Figure 3A) and NTA (Figure 3B). The electron microscopy and

particle size results showed that the ADSC-Ex had a double-layered membrane of approximately 80nm in diameter, with a cup-shaped structure. The nanoflow cytometry results showed that the positivity rate of the ADSC-Ex labeled with CD63 and CD9 was higher than that of the blank control group (Figure 3C).

3.4. Effect of ADSC-Ex on testicular injury models:

The results shown in Figure 4 revealed that the pathological results (Figure 4A) showed a significant decrease in the layer of germ cells and a sparse arrangement of the seminiferous tubules in the testicular tissue of the model group compared to the control group. The ADSCs cells and their ADSC-Ex significantly improved the testicular tissue damage, increased the number of germ cells, and improved the arrangement of the seminiferous tubules (Figure 4B-4D). The immunohistochemical results (Figure 5A and 5B) showed a significant decrease in the expression level of c-Kit in the model group compared to the control group, while the expression level of c-Kit in the testicular tissue was significantly increased after the intervention of ADSCs cells and their ADSC-Ex. The ELISA results (Figure 5C) showed a significant increase in the levels of malondialdehyde (MDA) in the model group compared to the control group, and a significant decrease in the levels of MDA in the ADSCs cells and their ADSC-Ex treated groups compared to the model group, with statistical significance ($P<0.05$). Similarly, testosterone (T) levels were significantly decreased in the model group compared to the control group, and both the treated groups rescued the decrease of T (Figure 5D).

4.Discussion:

In this study, we aimed to use adipose-derived mesenchymal stem cell-derived exosomes to treat rats with cisplatin-induced testicular injury. By using TEM, NTA, and nanoflow cytometry analysis, we successfully isolated and identified the ADSC-Ex. The results showed that the ADSC-Ex had a double-layered membrane of approximately 80nm in diameter, with a cup-shaped structure, and were positive for CD63 and CD9. The in vivo experiments demonstrated that the administration of ADSC-Ex significantly improved the testicular tissue damage, increased the number of germ cells, and improved the arrangement of the seminiferous tubules. The results of the immunohistochemical and ELISA analyses further supported the therapeutic effect of the ADSC-Ex.

Cisplatin-induced testicular injury is a well-known side effect of chemotherapy, which can lead to infertility and other reproductive problems (Shaaban et al., 2023). The mechanism of cisplatin-induced testicular injury is complex and involves oxidative stress, inflammation, and DNA damage (Negm et al., 2022). Cisplatin can induce the production of reactive oxygen species (ROS) and reduce the activity of antioxidant enzymes, leading to oxidative stress and damage to testicular cells (Abdel-Latif et al., 2022). In addition, cisplatin can activate the NF-κB signaling pathway and increase the expression of pro-inflammatory cytokines, leading to inflammation and tissue damage (Rahimi et al., 2022). Furthermore, cisplatin can directly damage DNA and induce apoptosis in testicular cells (Makled and Said, 2021).

Clinical studies have shown that cisplatin-induced testicular injury is common in cancer patients receiving chemotherapy, and can lead to a significant reduction in sperm count and motility (Hassanein et al., 2021). In addition, cisplatin-induced testicular injury can also lead to hormonal imbalances and other reproductive problems. Various strategies have been proposed to prevent or reduce cisplatin-induced testicular injury, including the use of antioxidants, hormone replacement therapy, and cryopreservation of sperm prior to chemotherapy (Abdel-Latif et al., 2022; Abdel-Wahab et al., 2021).

Basic research on cisplatin-induced testicular injury has focused on understanding the underlying mechanisms and developing new therapeutic strategies. Animal models have been used to study the effects of cisplatin on testicular function, and to evaluate the efficacy of potential treatments(Moradi et al., 2021). Recent studies have suggested that compounds such as melatonin, resveratrol, and curcumin may have protective effects against cisplatin-induced testicular injury by reducing oxidative stress and inflammation (Elrashidy and Hasan, 2020).

ADSC-Ex are small membrane-bound particles secreted by various cell types, including adipose-derived mesenchymal stem cells (ADSCs). ADSC-Ex plays a crucial role in intercellular communication, as they can transfer bioactive molecules, such as proteins, lipids, and nucleic acids, to recipient cells (Khandan-Nasab et al., 2023). In recent years, there has been a growing interest in the therapeutic potential of ADSC-Ex in various diseases, including

tissue injury, inflammation, and cancer (Taghavi et al., 2023).

ADSC-Ex has several advantages over ADSCs themselves, including lower immunogenicity, reduced risk of tumorigenesis, and easier storage and transportation (Feng et al., 2022). Moreover, ADSC-Ex can cross biological barriers, such as the blood-brain barrier, and target specific tissues or cells, making them an attractive therapeutic option (Guo et al., 2022). Several studies have investigated the therapeutic potential of ADSC-Ex in tissue injury models, such as myocardial infarction, acute kidney injury, and spinal cord injury (Hu et al., 2023). These studies have shown that ADSC-Ex can promote tissue repair, reduce inflammation, and improve functional recovery (Li et al., 2022).

In addition to tissue injury, ADSC-Ex has also been studied in cancer therapy. ADSC-Ex can deliver anti-tumor agents, such as miRNAs and chemotherapeutic drugs, to cancer cells and inhibit tumor growth (Liu et al., 2022). Moreover, ADSC-Ex can modulate the immune response and enhance the anti-tumor activity of immune cells (Lyu et al., 2022).

In our study, we isolated ADSCs from inguinal adipose tissue of rats. As previously reported (Meligy et al., 2019), we also used ultracentrifugation-based isolation technique to isolate double-layered membrane microvesicles of about 80nm in size, similar to "cup-shaped" exosomes, which is consistent with previous studies.

Chemotherapeutic drugs cannot distinguish between cancer cells and normal cells, thus causing damage to normal organs(Hou et al., 2005). Especially after

damaging the testis, it will have adverse effects on testicular tissue, blood, and semen parameters. This study confirms this view. At the same time, we also confirmed the damaging effect of cisplatin, a chemotherapeutic drug widely used in urological tumors (especially testicular cancer), on testicular tissue and germ cells, which is consistent with previous research conclusions (Gholami Jourabi et al., 2021; H and B, 2020).

Regarding the evaluation under an optical microscope, we found that testicular tissue exposed to cisplatin had obvious damage, manifested as a decrease in the thickness of the epithelium of the seminiferous tubules, a decrease in the diameter of the seminiferous tubules, and a large number of vacuoles in the interstitial cells. These changes caused a decrease in rat testicular spermatogenic function, similar to rats exposed to cisplatin and rats undergoing ischemia-reperfusion (Huang et al., 2018; Saral et al., 2016).

C-kit, as a stem cell factor receptor and a marker for distinguishing spermatogenic cell types, is strongly positive in all germ cells except for sperm cells. Our immunohistochemical results showed that the number of c-kit positive cells and the expression of all germ cells in the cisplatin exposure group were significantly decreased, which is consistent with previous research findings. However, treatment with ADSCs and exosomes significantly increased the number of c-kit positive cells (Oltulu et al., 2015).

The ROS induced by cisplatin can induce oxidative stress, causing damage to testicular tissue(Shaaban et al., 2023). This damage can lead to an imbalance

between pro-oxidants and antioxidants. Previous studies have found that MDA, as an oxidative stress marker, is produced by the peroxidation of lipids by ROS (Abdel-Latif et al., 2022). It affects the activity of mitochondrial respiratory chain complexes and key enzymes in the mitochondria in vitro. In this study, the increase of MDA in the cisplatin exposure group confirmed this finding (Rizk et al., 2018). The protective effect of ADSCs and their exosomes against oxidative stress can be attributed to the antioxidant capacity of stem cells through ROS clearance and the direct delivery of antioxidant enzyme mRNA or proteins by exosomes, thus protecting testicular tissue from oxidative stress, apoptosis, and promoting tissue regeneration.

Despite the promising results, several challenges need to be addressed before ADSC-Ex can be used in clinical setting (Peng et al., 2022). These include standardization of isolation and characterization methods, optimization of ADSC-Ex production and storage, and identification of the optimal dosing and delivery routes. Nevertheless, ADSC-Ex represents a promising therapeutic option with great potential for clinical translation.

5.Conclusion:

In this study, the ADSC-Ex were extracted and identified, and the effectiveness of adipose-derived stem cells and derived exosomes on testicular injury caused by cisplatin were proved.

Conflicts of Interest: The authors have no conflicts of interest to declare.

Data Availability: Data will be made available on request.

Ethical Statement: The authors are accountable for all aspects of the work in ensuring that questions related to the accuracy or integrity of any part of the work are appropriately investigated and resolved. The study was conducted in accordance with the Declaration of Helsinki (as revised in 2013). Experiments were performed under a project license(No.ZZU-LAC20190125) granted by the Animal Ethical and Welfare Committee, in compliance with the ethics committee of the First Affiliated Hospital of Zhengzhou University.

Acknowledgments:

Funding: This work was supported by the Henan Provincial Health Care Commission Provincial Ministry Project Fund (LHGJ20190276).

Abdel-Latif, R., Fathy, M., Anwar, H.A., Naseem, M., Dandekar, T., Othman, E.M., 2022. Cisplatin-Induced Reproductive Toxicity and Oxidative Stress: Ameliorative Effect of Kinetin. Antioxidants (Basel) 11.

Abdel-Wahab, B.A., Walbi, I.A., Albarqi, H.A., Ali, F.E.M., Hassanein, E.H.M., 2021. Roflumilast protects from cisplatin-induced testicular toxicity in male rats and enhances its cytotoxicity in prostate cancer cell line. Role of NF-kappaB-p65, cAMP/PKA and Nrf2/HO-1, NQO1 signaling. Food Chem Toxicol 151, 112133.

Elrashidy, R.A., Hasan, R.A., 2020. Stromal cell-derived factor-1alpha predominantly mediates the ameliorative effect of linagliptin against cisplatin-induced testicular injury in adult male rats. Cytokine 136, 155260.

Elsharkawi, M., Ghoneim, B., O'Sullivan, M., Lowery, A.J., Westby, D., Tawfick, W., Walsh, S.R., 2023. Role of Adipose Derived Stem Cells in Patients with Diabetic Foot Ulcers: Systematic Review and Meta-Analysis of Randomised Controlled Trials. Int J Low Extrem Wounds, 15347346231174554.

Feng, C.Y., Bai, S.Y., Li, M.L., Zhao, J.Y., Sun, J.M., Bao, H.J., Ren, Y., Su, X.M., 2022. Adipose-Derived Mesenchymal Stem Cell-Derived Exosomal miR-301a-3p Regulates Airway Smooth Muscle Cells During Asthma by Targeting STAT3. J Asthma Allergy 15, 99-110.

Gecys, D., Skredeniene, R., Gecyte, E., Kazlauskas, A., Balnyte, I., Jekabsone, A., 2023. Adipose Tissue-Derived Stem Cell Extracellular Vesicles Suppress Glioblastoma Proliferation, Invasiveness and Angiogenesis. Cells 12.

Gholami Jourabi, F., Yari, S., Amiri, P., Heidarianpour, A., Hashemi, H., 2021. The ameliorative effects of methylene blue on testicular damage induced by cisplatin in rats. Andrologia 53, e13850.

Guo, G., Tan, Z., Liu, Y., Shi, F., She, J., 2022. The therapeutic potential of stem cell-derived exosomes in the ulcerative colitis and colorectal cancer. Stem Cell Res Ther 13, 138.

H, A.A.A., B, G.E., 2020. Cisplatin induced testicular damage through mitochondria mediated apoptosis, inflammation and oxidative stress in rats: impact of resveratrol. Endocr J 67, 969-980.

Hassanein, E.H.M., Abdel-Wahab, B.A., Ali, F.E.M., Abd El-Ghafar, O.A.M., Kozman, M.R., Sharkawi, S.M.Z., 2021. Trans-ferulic acid ameliorates cisplatin-induced testicular damage via suppression of TLR4, P38-MAPK, and ERK1/2 signaling pathways. Environ Sci Pollut Res Int 28, 41948-41964.

Hou, M., Chrysis, D., Nurmio, M., Parvinen, M., Eksborg, S., Soder, O., Jahnukainen, K., 2005. Doxorubicin induces apoptosis in germ line stem cells in the immature rat testis and amifostine cannot protect against this cytotoxicity. Cancer Res 65, 9999-10005.

Hu, Y.C., Zhang, X.B., Lin, M.Q., Zhou, H.Y., Cong, M.X., Chen, X.Y., Zhang, R.H., Yu, D.C., Gao, X.D., Guo, T.W., 2023. Nanoscale Treatment of Intervertebral Disc Degeneration: Mesenchymal Stem Cell Exosome Transplantation. Curr Stem Cell Res Ther 18, 163-173.

Huang, X., Ding, J., Li, Y., Liu, W., Ji, J., Wang, H., Wang, X., 2018. Exosomes derived from PEDF modified adipose-derived mesenchymal stem cells ameliorate cerebral ischemia-reperfusion injury by regulation of autophagy and apoptosis. Exp Cell Res 371, 269-277.

Khandan-Nasab, N., Mahdipour, E., Askarian, S., Kalantari, M.R., Ramezanian, N., Kazemi Oskuee, R., 2023. Design and characterization of adipose-derived mesenchymal stem cell loaded alginate/pullulan/hyaluronic acid hydrogel scaffold for wound healing applications. Int J Biol Macromol 241, 124556.

Kohsaka, T., Minagawa, I., Morimoto, M., Yoshida, T., Sasanami, T., Yoneda, Y., Ikegaya, N., Sasada, H., 2020. Efficacy of relaxin for cisplatin-induced testicular dysfunction and epididymal spermatotoxicity. Basic Clin Androl 30, 3.

Li, C., An, Y., Sun, Y., Yang, F., Xu, Q., Wang, Z., 2022. Adipose Mesenchymal Stem Cell-Derived Exosomes Promote Wound Healing Through the WNT/beta-catenin Signaling Pathway in Dermal Fibroblasts. Stem Cell Rev Rep 18, 2059-2073.

Li, W., Chen, X., Zou, F., He, X., 2023. Extracellular Vesicles Derived from Hypoxia Treated Human Adipose Stem Cells Increase Proliferation and Angiogenic Differentiation in Human Adipose Stem Cells. Aesthet Surg J.

Liu, T., Li, T., Zheng, Y., Xu, X., Sun, R., Zhan, S., Guo, X., Zhao, Z., Zhu, W., Feng, B., Wei, F., Jiang, N., Wang, J., Chen, X., Fang, F., Guo, H., Yang, R., 2022. Evaluating adipose-derived stem cell exosomes as miRNA drug delivery systems for the treatment of bladder cancer. Cancer Med 11, 3687-3699.

Lyu, K., Liu, T., Chen, Y., Lu, J., Jiang, L., Liu, X., Liu, X., Li, Y., Li, S., 2022. A "cell-free treatment" for tendon injuries: adipose stem cell-derived exosomes. Eur J Med Res 27, 75.

Makled, M.N., Said, E., 2021. Tranilast abrogates cisplatin-induced testicular and epididymal injuries: An insight into its modulatory impact on apoptosis/proliferation. J Biochem Mol Toxicol 35, e22817.

Meligy, F.Y., Abo Elgheed, A.T., Alghareeb, S.M., 2019. Therapeutic effect of adipose-derived mesenchymal stem cells on Cisplatin induced testicular damage in adult male albino rat. Ultrastruct Pathol 43, 28-55.

Moradi, M., Goodarzi, N., Faramarzi, A., Cheraghi, H., Hashemian, A.H., Jalili, C., 2021. Melatonin protects rats testes against bleomycin, etoposide, and cisplatin-induced toxicity via mitigating nitro-oxidative stress and apoptosis. Biomed Pharmacother 138, 111481.

Negm, W.A., El-Kadem, A.H., Hussein, I.A., Alqahtani, M.J., 2022. The Mechanistic Perspective of Bilobetin Protective Effects against Cisplatin-Induced Testicular Toxicity: Role of Nrf-2/Keap-1 Signaling, Inflammation, and Apoptosis. Biomedicines 10.

Oltulu, F., Aktug, H., Uysal, A., Turgan, N., Oktem, G., Erbas, O., Yavasoglu, N.K., Yavasoglu, A., 2015. Immunoexpressions of embryonic and nonembryonic stem cell markers (Nanog, Thy-1, c-kit) and cellular connections (connexin 43 and occludin) on testicular tissue in thyrotoxicosis rat model. Hum Exp Toxicol 34, 601-611.

Peng, L., Chen, Y., Shi, S., Wen, H., 2022. Stem cell-derived and circulating exosomal microRNAs as new potential tools for diabetic nephropathy management. Stem Cell Res Ther 13, 25.

Phelps, J., Leonard, C., Shah, S., Krawetz, R., Hart, D.A., Duncan, N.A., Sen, A., 2022. Production of Mesenchymal Progenitor Cell-Derived Extracellular Vesicles in Suspension Bioreactors for Use in Articular Cartilage Repair. Stem Cells Transl Med 11, 73-87.

Qin, Y., Ge, G., Yang, P., Wang, L., Qiao, Y., Pan, G., Yang, H., Bai, J., Cui, W., Geng, D., 2023. An Update on Adipose-Derived Stem Cells for Regenerative Medicine: Where Challenge Meets Opportunity. Adv Sci (Weinh), e2207334.

Rahimi, A., Asadi, F., Rezghi, M., Kazemi, S., Soorani, F., Memariani, Z., 2022. Natural products against cisplatin-induced male reproductive toxicity: A comprehensive review. J Biochem Mol Toxicol 36, e22970.

Rizk, H., Tohamy, A.F., Sayed, W.M., Prince, A., 2018. Ameliorative effects of bone marrow derived pancreatic progenitor cells on hyperglycemia and oxidative stress in diabetic rats. Acta Histochem 120, 412-419.

Saral, S., Ozcelik, E., Cetin, A., Saral, O., Basak, N., Aydin, M., Ciftci, O., 2016. Protective role of Diospyros lotus on cisplatin-induced changes in sperm characteristics, testicular damage and oxidative stress in rats. Andrologia 48, 308-317.

Shaaban, A.A., Zaghloul, R.A., Kafl, H.E., El-Kashef, D.H., 2023. Ameliorative effect of desloratadine against cisplatin-induced renal and testicular toxicity in rats: Attention to TLR4/NLRP3 inflammasome signaling pathway. Life Sci 316, 121441.

Simao, V.A., Brand, H., da Silveira-Antunes, R.N., Fukasawa, J.T., Leme, J., Tonso, A.,

Ribeiro-Paes, J.T., 2023. Adipose-derived stem cells (ASCs) culture in spinner flask: improving the parameters of culture in a microcarrier-based system. Biotechnol Lett.

Taghavi, S., Abdullah, S., Shaheen, F., Packer, J., Duchesne, J., Braun, S.E., Steele, C., Pociask, D., Kolls, J., Jackson-Weaver, O., 2023. Exosomes and Microvesicles From Adipose-derived Mesenchymal Stem Cells Protects the Endothelial Glycocalyx From LPS Injury. Shock.

Wang, G., Li, H., Long, H., Gong, X., Hu, S., Gong, C., 2022. Exosomes Derived from Mouse Adipose-Derived Mesenchymal Stem Cells Alleviate Benzalkonium Chloride-Induced Mouse Dry Eye Model via Inhibiting NLRP3 Inflammasome. Ophthalmic Res 65, 40-51.

Xia, L., Zhang, C., Lv, N., Liang, Z., Ma, T., Cheng, H., Xia, Y., Shi, L., 2022. AdMSC-derived exosomes alleviate acute lung injury via transferring mitochondrial component to improve homeostasis of alveolar macrophages. Theranostics 12, 2928-2947.

 Click here to access/download;Manuscript;figure legends.docx

Figure 1: Results of models validation. A: showed a significant decrease in the layer of germ cells and a sparse arrangement of the seminiferous tubules in the testicular tissue of the model group compared to the control group. B: showed a significant decrease in the expression level of c-Kit in the model group compared to the control group. C: showed a significant increase in the levels of follicle-stimulating hormone (FSH) and luteinizing hormone (LH), a significant decrease in the level of testosterone (T).

Figure 2: Results of ADSC-Ex: A: showed positive expression of CD29 and CD90 and negative expression of CD11b, CD34, and CD45 in the cells; B: Results of Alizarin Red staining.

Figure 3: Results of evaluation of ADSC-Ex. A-B: morphology and size of ADSC-Ex were evaluated using TEM and NTA. C: Positivity rate of the ADSC-Ex labeled with CD63 and CD9 was higher than that of the blank control group.

Figure 4: Histopathological evaluation of the effect of ADSC-Ex on testicular injury models. A: results of histopathology, H&E stained sections in the testis of rat, (a,c) for (G I), (b,f) for (G II), (c,g) for (G III), (d,h) for (G IV) , showing : **a** seminiferous tubules (st) , interstitial tissue (i) and blood capillary (bc) , **b** large interstitial vacuoles (v) , distorted and reduced seminiferous tubules(*), **e** spermatogonia (G), sperm (sp), **f** Irregular and deformed seminiferous tubule epithelium(*),sloughing of degenerated spermatogenic cells(#), **g** restoration of epithelium thickness (arrow), **h** Restoration of the normal architecture of

seminiferous tubule.B-D: changes of seminiferous tubules epithelium, diameter and testicular biopsy scores.

Figure 5: Effect of ADSC-Ex on testicular injury models. A-B: immunohistochemical results. C-D: changes of MDA content and T content.

Click here to access/download;Figure;figure.1.tif

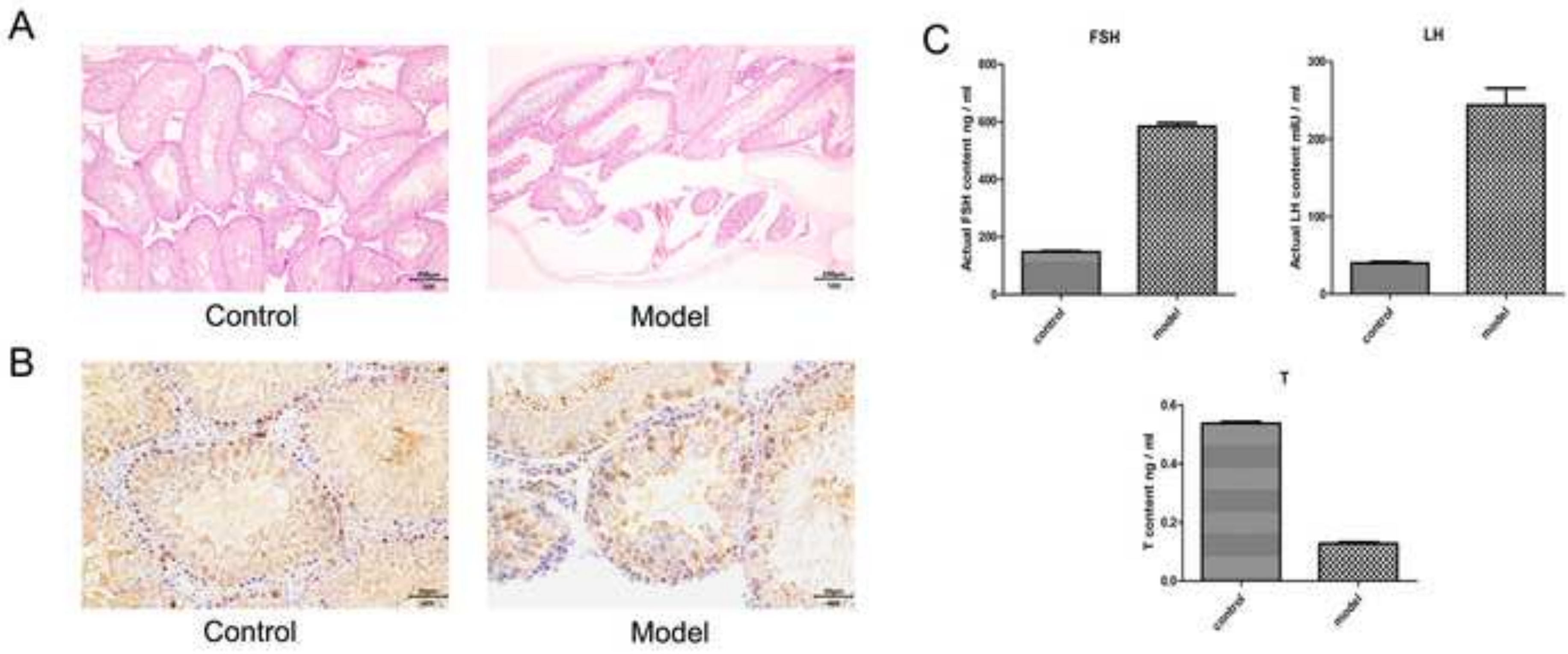

Figure Click here to access/download;Figure;figure.2.tif

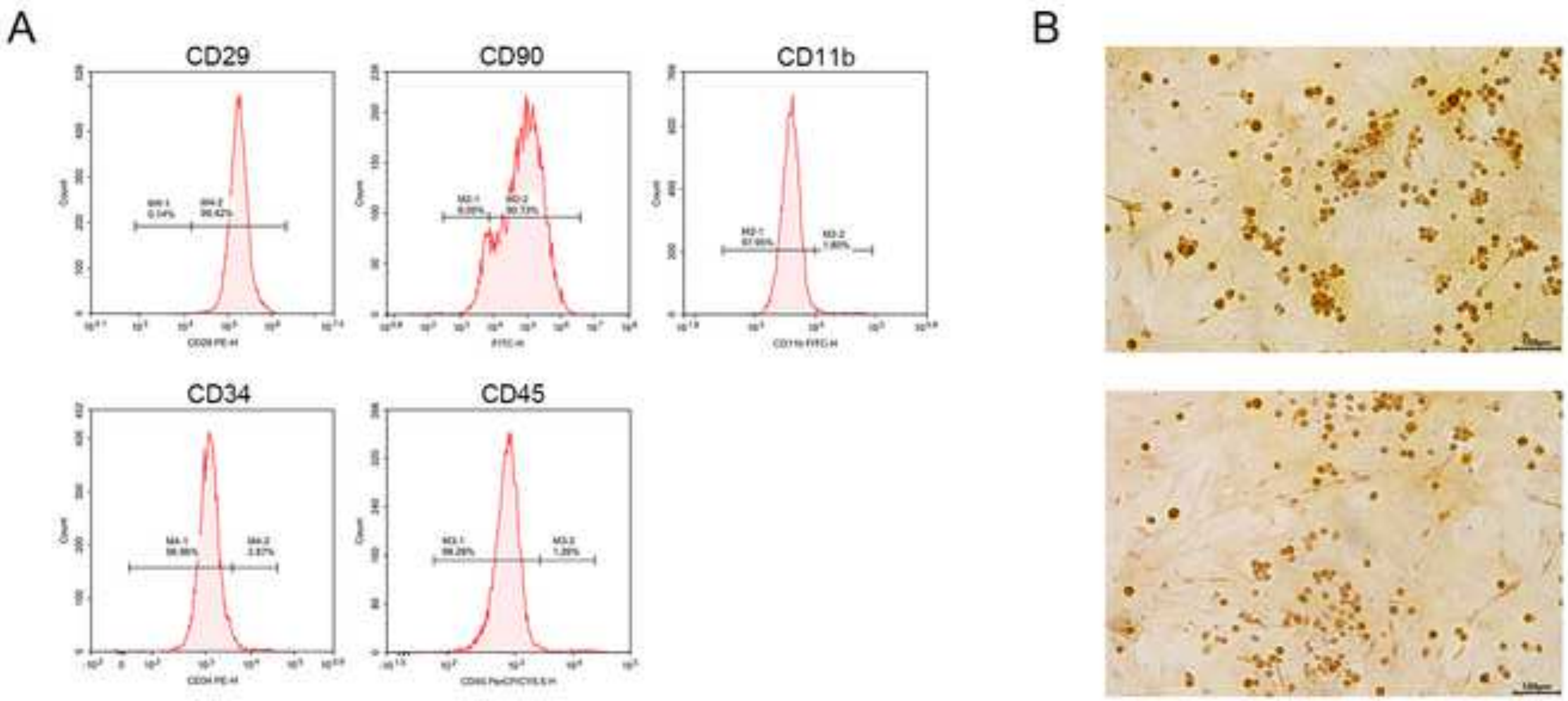

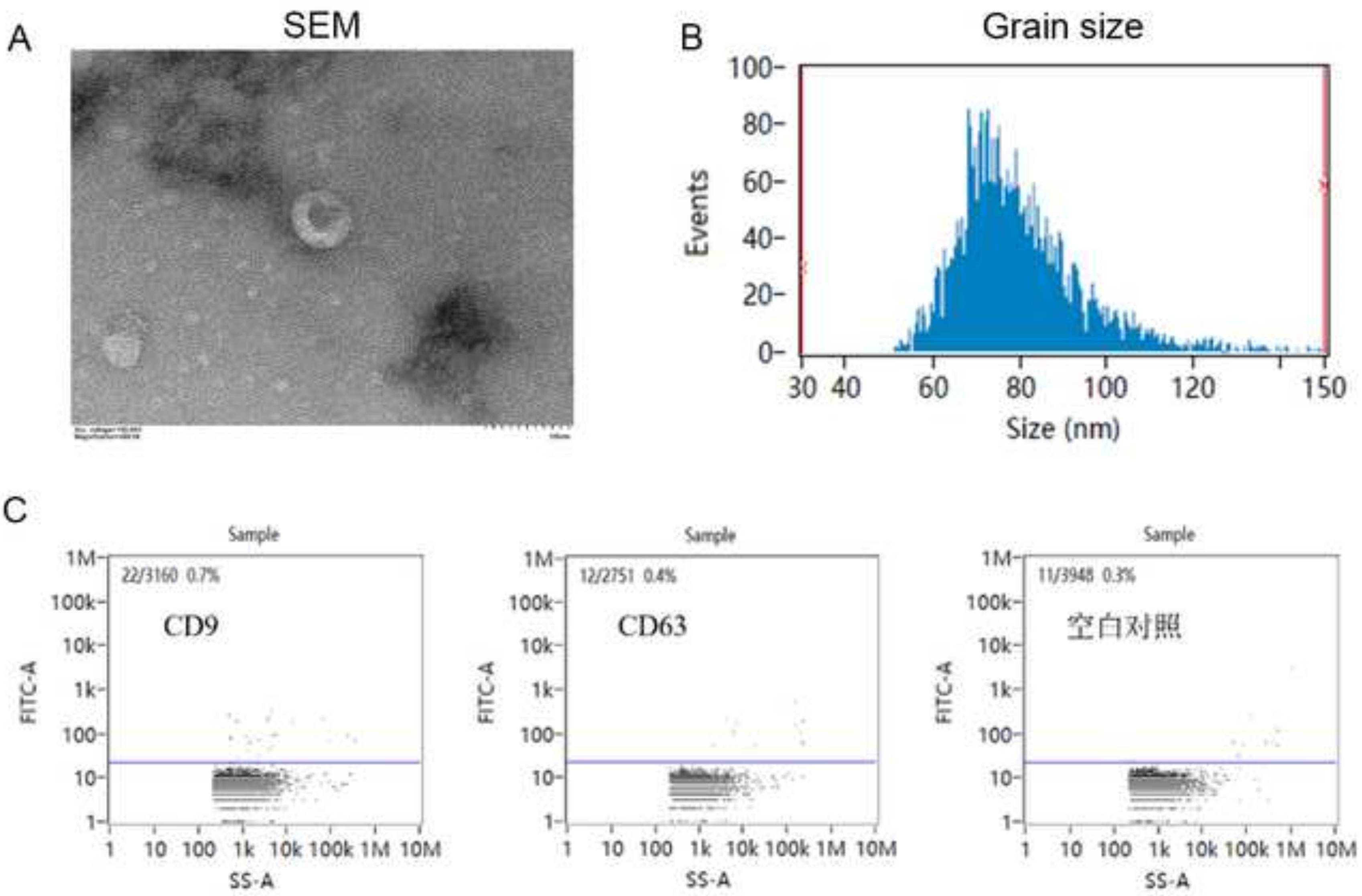
A
SEM
B
Grain size
Events
100
80
60
40
20
0
30 40 60 80 100 120 150
Size (nm)
C
Sample
1M
100k
10k
1k
100
10
1
22/3160 0.7%
CD9
12/2751 0.4%
CD63
11/3948 0.3%
空白对照
FITC-A
1 10 100 1k 10k 100k 1M 10M
SS-A

Click here to access/download;Figure;figure.4.tif

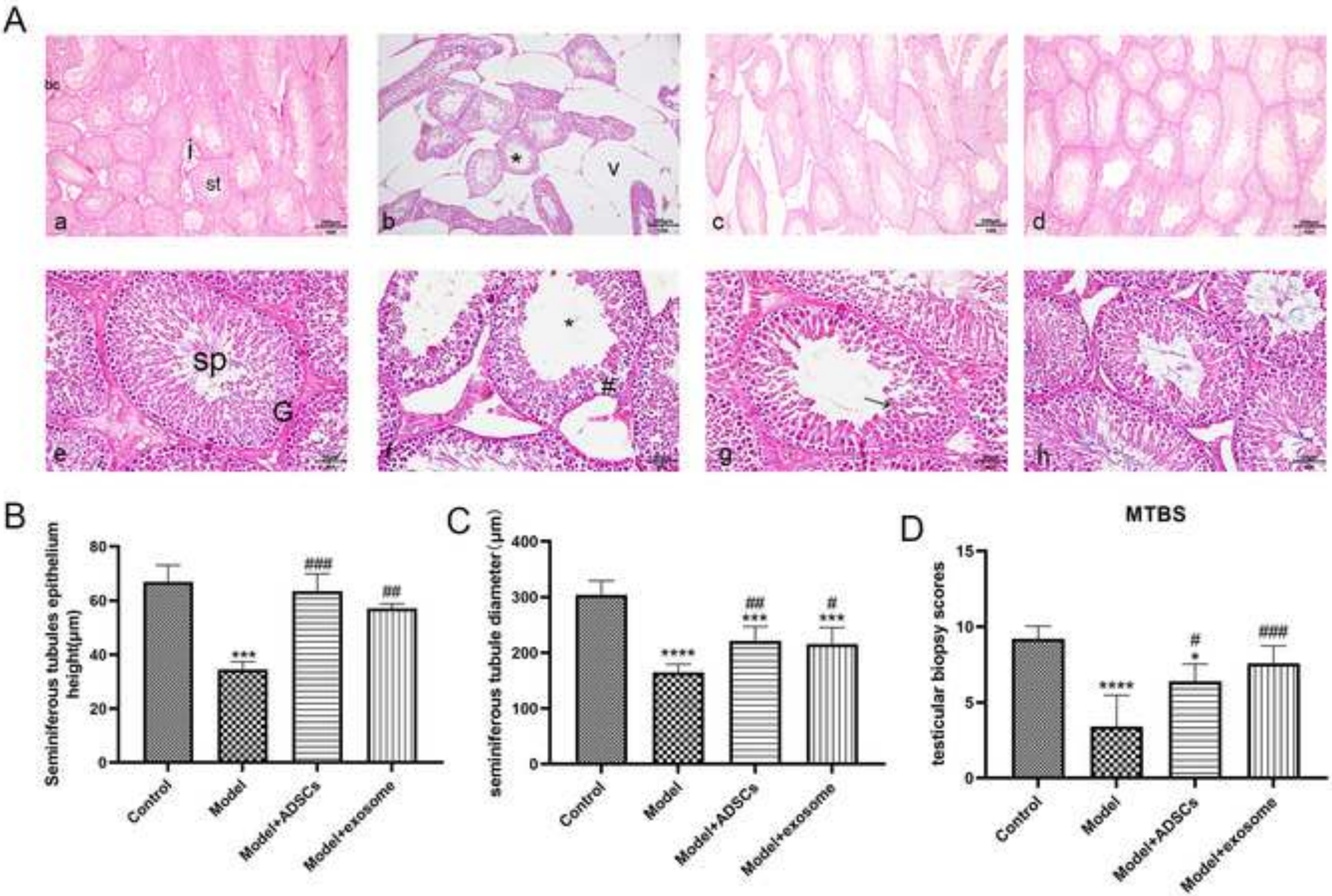

Figure Click here to access/download;Figure;figure.5.tif

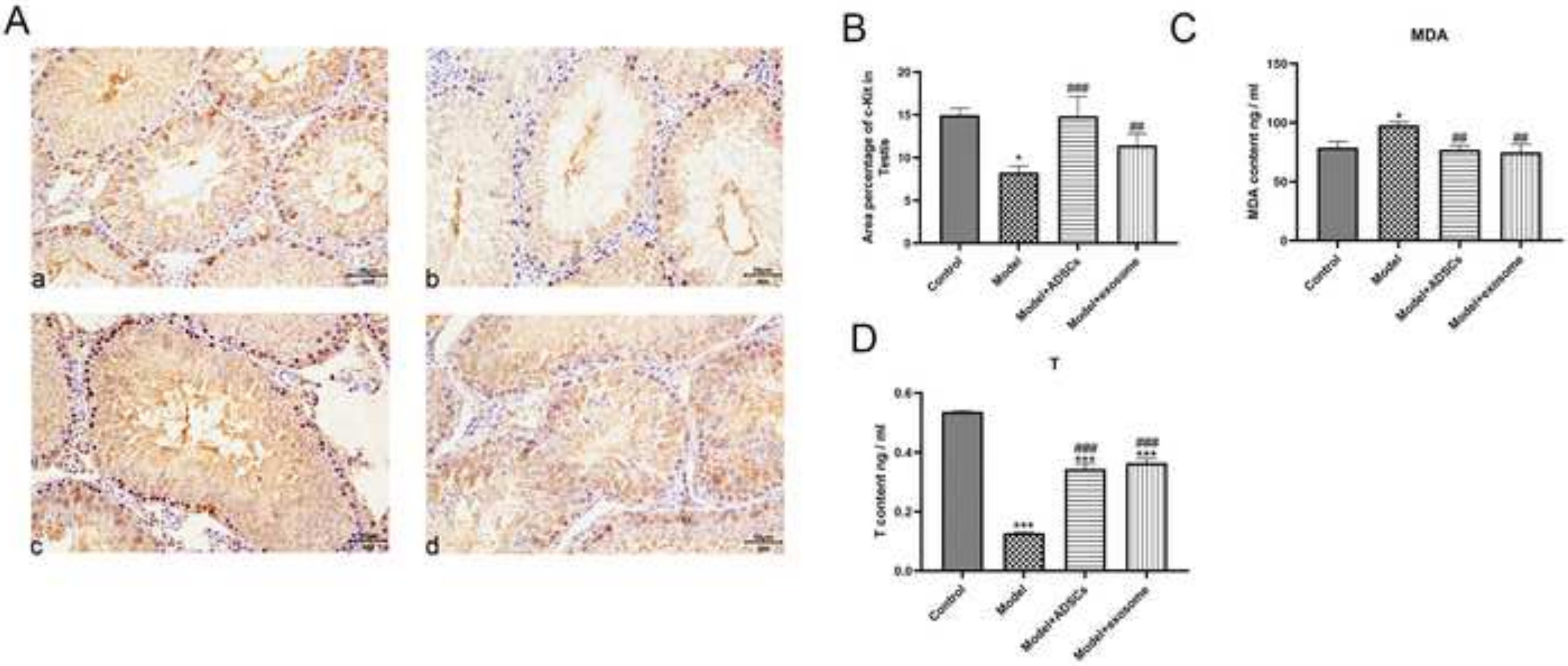

Click here to access/download;Figure;Table1.tif

Testicular biopsy scores (MTBS)

Score	Description
1	No cells
2	Sertoli cells without germ cells
3	Only spermatogonia
4	Only a few spermatocytes
5	Many spermatocytes
6	Only a few early spermatids
7	Many early spermatids without differentiation
8	Few late spermatids
9	Many late spermatids
10	Full spermatogenesis
